# La campagne s'urbanise: résultats d'un dépistage du diabète et des dysglycémies dans une zone rurale de la région de Marrakech

**DOI:** 10.11604/pamj.2014.19.31.3690

**Published:** 2014-09-13

**Authors:** Laila Ennazk, Ghizlane El M'ghari, Nawal El Ansari

**Affiliations:** 1 Service d'Endocrinologie Diabétologie et des Maladies métaboliques, CHU MedVI, Laboratoire PCIM, Faculté de Médecine et de Pharmacie Marrakech, Université Cadi Ayad, Marrakesh, Maroc

**Keywords:** Diabète, dépistage, hypertension artérielle, obésité, milieu rural, Maroc, diabetes, screening, High blood pressure, obesity, rural area, Morocco

## Abstract

**Introduction:**

Le diabète constitue un problème de santé publique au Maroc. L'augmentation de sa prévalence se fait en parallèle avec l'augmentation des chiffres de l'obésité et de l'hypertension artérielle (HTA). Les facteurs environnementaux dont l'urbanisation influencent avec force cet état des lieux. En milieu rural, la tendance tend à s'inverser du fait du changement des habitudes de vie. Notre travail avait comme but de dépister la dysglycémie dans une commune rurale de la région de Marrakech.

**Méthodes:**

Ce travail de dépistage fait sur une journée a concerné une population tout-venant de 200 personnes. La glycémie capillaire à jeun, la tension artérielle, le poids, la taille, l'indice de masse corporelle et le tour de taille ont été mesurés pour toutes les personnes qui se sont présentées.

**Résultats:**

Deux cent sujets ont été examinés. 31% des sujets examinés étaient diabétiques. Chez les non diabétiques, 57,9% avaient une glycémie capillaire à jeun au-delà de 1,1g/l, 51,2% entre 1,1 g/l et 1,26 g/l et 48,7% au-delà de 1,26 g/l. Sur le plan tensionnel, 95% des patients examinés n'étaient pas connus hypertendus. L'obésité androïde touche 63,7% des patients examinés.

**Conclusion:**

Les chiffres retrouvés lors de cette caravane médicale sont supérieurs à nos attentes. L'urbanisation du milieu rural a un impact sur le profil métabolique et tensionnel de cette population; ce qui devrait impliquer une démarche préventive de santé publique.

## Introduction

La prévalence des maladies non transmissibles (MNT) est en augmentation alarmante de part le monde. Prêt des deux tiers des décès annuels sont dus au MNT principalement les maladies cardiovasculaires liés au diabète et à l'hypertension artérielle (HTA) [1]. Ces derniers, longtemps considérés comme des maladies des pays industrialisés, menacent de plus en plus les pays en voies de développement (PVD) qui vivent pleinement la transition épidémiologique. Le mode de vie, l'urbanisation, et les facteurs comportementaux influencent avec force cet état des lieux. Près de 85% des diabètes non diagnostiqués existent dans les PVD où le manque de ressources ne permet pas de prioriser une politique de dépistage du diabète et des maladies non transmissibles [2]. Au Maroc, le diabète représente un problème de santé publique. Sa prévalence actuelle est aux alentours de 10% [3]. Nous avons organisé en collaboration avec l'association des médecins internes de Marrakech une journée de dépistage du diabète, des dysglycémies dans la commune rurale de « Saada » dans la région de Marrakech.

## Méthodes

L'examen des patients et le recueil des donnés s'est fait sur une journée. Onze postes ont accueilli les patients: 5 postes pour la mesure de la glycémie capillaire, 3 postes pour la mesure de la pression artérielle, 3 pour la mesure du périmètre ombilicale, du poids et de la taille. Une fiche est remplie au fur et à mesure comprenait l’âge, le sexe, les antécédents, la glycémie capillaire à jeun faite après 8 heures de jeun, les données de la bandelette urinaire et le périmètre ombilical. La glycémie capillaire était mesurée et recontrôlée une deuxième fois si le premier chiffre était supérieur à 1,26 g/l à jeun. A été considéré comme GCAJ élévée une mesure au-delà de 1,1g/l. La bandelette urinaire est faite au-delà d'un chiffre de glycémie capillaire de 1,80d/l. La PA a été prise au repos, manuellement au brassard, et a été recontrolée si une première mesure est au-delà de 140/90. Le périmètre ombilical était mesuré pour toute la population. Le surpoids a été défini par un IMC entre 25 et 30 Kg/m^2^, est une obésité par un IMC dépassant 30 Kg/m^2^. Les chiffres de TA on été classés selon 3 catégories:HTA stade 1: de 140/90-159/99 mm Hg; HTA stade 2: de 160/100 à 179/109 mm Hg; HTA stade 3: de 180/110 mm Hg et plus. Les personnes ayant des chiffres anormaux étaient dirigées vers trois postes de consultations afin d'insister sur les mesures hygiéno-diététiques, prescrire le traitement adapté et adresser le malade en consultation. L'analyse statistique est faite par le logiciel SPSS version 17.0.

## Résultats

Le nombre de patients dépistés était de 200, dont 130 femmes (soit 65,3%) et 70 hommes (soit 34,7%). Les âges étaient entre 16 et 88 ans avec un âge moyen de 52,73 ± 12,84. Les tranches d’âge se répartissaient comme suite: 8 personnes avaient un âge compris entre 15 et 30 ans soit 4%. 133 personnes avaient un âge compris entre 30 et 60 ans soit 82%. Cent quarante deux des GCAJ mesurées étaient au-delà de 1,1g/l (71%). 62 personnes étaient connu porteuses d'un diabète (31%). Parmi les 138 non diabétiques, 57,9% soit 80 personnes présentaient une GCAJ au-delà de 1,1g/l dont 54 femmes (67,5%) et 26 hommes (32,5%). Chez ces derniers, 38 (51,2%) personnes avaient une GCAJ entre 1,1 g/l et 1,26 g/l. Alors que 42 personnes (48,7%) personnes avaient une glycémie capillaire au-delà de 1,26 g/l recontrôlée à deux reprises. Sur les 62 patients diabétiques, 56,8% avaient une pression artérielle inferieure à 140/90mmgh. 60,5% d'entre eux avaient un âge entre 30 et 60 ans et 34,2% dépassaient les 60 ans. Chez les patients porteurs d'une hyperglycémie modérée à jeun, 39% avait un surpoids et 34,4% avaient une obésité. Par contre chez ceux ayant une glycémie au-delà de 1,26g/l, 25,6% avaient un surpoids et 51,2% avait une obésité. Les IMC et les tours de tailles sont représentés dans le Tableau 1. L'obésité androïde touche 127 (63,7%) des personnes dépistées. L'obésité touchait 10% des hommes et 41,1% des femmes. Concernant l'IMC, nous avons retrouvé 41,5% de personnes en surpoids, 37,5% ayant une obésité et 21,0% ayant un IMC normal. Nous avons retrouvé une TA au-delà de 140/90mmhg chez 44,5% des patients. Le dépistage a retrouvé des chiffres tensionnels élevés chez 68 patients non connus hypertendus (44,2%). Trois catégories ont été identifiées (Tableau 2): HTA stade 1: 57 soit 28,2% dont 47 de novo (soit 82,8%); HTA stade 2: 21 soit 11,6% dont 18 de novo (soit 76,9%); HTA stade 3: 11 soit 5,7% dont 6 de novo (soit 50%). Les hypertendus connus et traités étaient au nombre de 7 soit 17,5% des hypertendus recensés et 3,5% des personnes recensées. 0,3% des hypertendus connus traités ont une PA < 140/90 mm Hg.

**Tableau 1:** Caractéristiques démographiques et métaboliques des sous groupes connus diabétiques et non connus diabétiques

	Connu diabétique N=62 (31%)	Non connu diabétique N=138 (69%)
**SEXE**		
F	44 (71,0)	87 (62,7)
M	18 (28,9)	51 (32,2)
**AGE**		
15-30	3 (5,2)	5 (3,4)
30-60	38 (60,5)	95 (68,6)
>60	21 (34,2)	38 (27,9)
**IMC**		
Normal	10 (15,7)	32 (23,2)
Surpoids	29 (47,3)	54 (39,5)
obésité	23 (36,8)	51 (37,2)
**Tour de taille**		
Homme >102	6 (9,6)	39 (28,2)
Homme <102	12(19,3)	12 (8,6)
Femme >88	12 (19,3)	39 (28,2)
Femme <88	32 (51,6)	48 (34,7)
**GCAJ**		
<1,1	10 (16,1)	58 (42,0)
1,1-1,26	44 (70,9)	38(27,5)
>1,26	8 (12,9)	42 (30,4)

**Tableau 2:** Les chiffres tensionnels retrouvés chez les diabétiques et les non diabétiques

	Connus diabétiques N=62 (31%)	Non connus diabétique N=138 (69%)
HTA stade 1	15 (24,3)	42 (30,5)
HTA stade 2	8 (13,5)	13 (9,4)
HTA stade 3	3 (5,4)	8 (5,8)

## Discussion

La prévalence des maladies non transmissibles tels le diabète et l'HTA est en augmentation alarmante dans les pays en voie de développement. Ceci est dû à l'augmentation de la prévalence du surpoids et de l'obésité qui, durant les dix dernières années se sont ajoutés à la malnutrition et aux maladies infectieuses. Ceci majore le coût de santé pour ces pays aux ressources limitées qui vivent pleinement leur transition épidémiologique. Durant les 20 dernières années, l'obésité a triplé dans les pays en voie de développement qui adoptent de plus en plus le mode de vie occidental. Ainsi, la sédentarité et le changement des habitudes alimentaires ont un impact direct sur l'augmentation de cette prévalence. Le moyen orient, les îles pacifiques, l'Asie du Sud-est et la Chine semblent être les plus menacés [4]. Au Maroc, une enquête nationale a estimé la prévalence de l'obésité à 13,5% [5]. La première étude observationnelle de l'obésité faite dans la région de Marrakech en 2008 a rapporté à l'époque un chiffre de 21% [6]. Les chiffres que nous avons retrouvés sont ainsi supérieurs à nos attentes. Cette large proportion d'obésité dans la population examinée explique les chiffres élevés de dys-glycémies et d'HTA. Le nombre de dysglycémies retrouvée lors de cette journée de dépistage est de 57,9% parmi les patients non connus diabétiques. Ces chiffres, qui sont supérieurs à nos attentes semblent être supérieurs également au chiffres retrouvés lors de ce type de campagnes sanitaires. Une campagne de dépistage faite à Brazzaville avait relevé seulement 8,8% de chiffres glycémiques au-delà des objectifs définis [7]. Nous notons que les dysglycémies enregistrées étaient plus fréquentes chez les femmes (67,5%) et que une GCAJ supérieure à 1,26 g/l était plus prévalente chez la tranche d’âge entre 30 et 60 ans. Il est important de pondérer ces chiffres, puisque nous nous sommes basés sur des mesures de glycémies capillaires. En effet, l'estimation de la glycémie par lecteur était le moyen le plus adapté à ce type de travail d'une journée. Nous reconnaissons également l'incrustation d'un biais de sélection puisque la population été au courant qu'il s'agissait d'une campagne de dépistage. D'autre part, l'étude de l'impact du lieu de résidence sur le diabète et obésité devrait prendre en considération la durée totale d'exposition au milieu urbain ou rural [8] et le niveau socio-économique [9] qui n'ont pas été détaillés chez nos patients.

La proportion de chiffres tensionnels élevée retrouvée est importante. Les trois quart des hypertendus dans le monde vivent dans les pays en voies de développement où la maladie est sous diagnostiquée et difficilement contrôlée [10]. Nous avons trouvé le même chiffre d'HTA mal contrôlée dans une zone rurale en Ecuador où le taux de contrôle était de 0,3% [11]. Les conditions de prise de tension artérielle ne nous permettent pas de poser le diagnostique d'HTA chez les patients dépistés, il n'en reste pas moins que 16% des patients avaient une pression artérielle supérieure à 160/100 mmHg. De plus, nous estimons que l'annonce d'un chiffre tensionnel élevé aurait certainement un impact sur la perception de la maladie et sur l'adhérence aux règles hygiéno-diététiques et thérapeutiques, surtout avec la difficulté d'accès au soin que connaît cette population rural où le dispensaire n'assure que des prestations de soin de base fournie par un seul infirmier.

La majorité de personnes dépistées (63,7%) présentait une obésité androïde. Les facteurs comportementaux, nutritionnels ont certainement un impact sur cet état des lieux. Dans notre contexte, nous ne nions pas également l'influence de facteurs culturels car au Maroc comme dans d'autres pays d'Afrique l'obésité est un signe de beauté. L'étude des causes à l'origine de l'augmentation de l'incidence de l'obésité, du diabète et de l'HTA à savoir la pauvreté, les inégalités sociales, le vieillissement de la population et le changement des habitudes alimentaires, permettent de mieux planifier l'approche préventive. La revue de la littérature montre que la prévention du diabète et de ses complications avait un impact considérable en terme de coût [12]. Certains auteurs ont proposé une approche préventive bien en amont de l'acte de soin, visant à promouvoir l'état nutritionnel et le mode de vie chez des parents jeunes avant même la conception. Une approche vraisemblablement adaptée au pays en voie de développement [13].

## Conclusion

Ce travail de dépistage fait sur une journée a permis de mettre le point sur les données en milieu rural concernant l'obésité, le diabète et l'HTA et qui sont directement liés à la morbi-mortalité cardiovasculaire. Les chiffres que nous avons trouvés dévoilent une réalité marocaine; c'est que à l'occidentalisation du mode de vie vient se chevaucher l'urbanisation du monde rural. Le coût énorme de ces maladies nous pousse à planifier des mesures préventives dans une véritable démarche de santé publique.
